# Relationship Between Serum Magnesium Levels and Glycemic Control in Type 2 Diabetes Mellitus: A Case-Control Study

**DOI:** 10.7759/cureus.117718

**Published:** 2026-10-10

**Authors:** Shahina Abdul Rahiman, Ganga Sanjeev, Gayathri Prathap, Gayathry Rajasekharan, Rajasekharan Chandrasekharan

**Affiliations:** 1 Internal Medicine, Azeezia Institute of Medical Sciences and Research, Kollam, IND; 2 Internal Medicine, Sree Uthradom Thiurnal (SUT) Academy of Medical Sciences, Thiruvananthapuram, IND; 3 Pathology, Government Medical College, Thrissur, IND

**Keywords:** hba1c, hypomagnesemia, insulin resistance, magnesium, type 2 diabetes mellitus

## Abstract

Introduction: Type 2 diabetes mellitus (T2DM) is a prevalent metabolic disorder that represents a global health burden. Recent findings indicate that serum magnesium plays a crucial role in glucose metabolism and insulin function. Insufficient magnesium levels are associated with diminished insulin secretion and increased insulin resistance. However, region-specific data from South India, particularly Kerala, remain limited.

Materials and methods: A case-control study was conducted in the Department of Medicine of a tertiary care hospital in Kerala from November 2022 to March 2024 to compare serum magnesium levels between adults with uncontrolled and controlled T2DM and to assess the associations of serum magnesium levels with HbA1c and other clinical and biochemical variables. A total of 34 patients with uncontrolled T2DM (HbA1c >7%) and 34 patients with controlled T2DM (HbA1c ≤7%), classified according to the 2021 American Diabetes Association glycemic target guidelines, were matched by age and sex. Demographic and laboratory data were analyzed using SPSS Statistics version 25 (IBM Corp. Released 2017. IBM SPSS Statistics for Windows, Version 25.0. Armonk, NY), with p < 0.05 considered statistically significant.

Results: Demographic characteristics were similar across groups. Uncontrolled cases had significantly higher HbA1c (median 8.3%, IQR 7.83-9.0 vs 6.7%, IQR 6.5-6.9; p < 0.001), fasting blood sugar (median 187.5, IQR 142-204 vs 120 mg/dL, IQR 110.25-131.75; p < 0.001), and blood pressure than controls. Hypomagnesemia occurred only in patients with uncontrolled T2DM (5/34, 14.7%); median serum magnesium was significantly lower in cases than in controls (2.1, IQR 1.9-2.3 vs 2.3 mg/dL, IQR 2.1-2.3; p = 0.018). Within cases, serum magnesium correlated inversely with triglycerides (ρ = −0.417, p = 0.014) but not significantly with HbA1c (ρ = −0.196, p = 0.266).

Conclusions: Serum magnesium was lower in uncontrolled than in controlled T2DM, with hypomagnesemia only in the uncontrolled group. Within cases, magnesium correlated inversely with triglycerides but not significantly with HbA1c. Causality cannot be established given the design; larger prospective and interventional studies are needed.

## Introduction

Diabetes mellitus (DM) includes a group of metabolic disorders marked by high blood glucose levels. In 2017, approximately 451 million adults aged 18-99 lived with diabetes, and projections indicate that this number will rise to 693 million by 2045 [1]. South Asia, home to nearly a quarter of the world's population, is experiencing a significant increase in noncommunicable diseases, such as diabetes. As the region's largest country, India is especially affected by this trend. Because conditions vary across Indian states, national averages do not reflect regional differences [2]. Despite advanced health indicators, Kerala has seen a notable rise in type 2 DM (T2DM) across all demographic groups. This aligns with the "Asian paradox," where high rates of metabolic disorders occur despite overall health improvements [3].

Even with progress in diagnosis and treatment, 76.6% of Indians with T2DM have poor glycemic control [4]. A major contributing factor is the "thin-fat" phenotype observed in many Indians, in which a lean appearance masks excess adiposity and reduced muscle mass. This condition is exacerbated by abnormalities in glucose and lipid metabolism, abdominal obesity, and hypertension, all of which contribute to insulin resistance. Indians also tend to develop T2DM at a younger age, speeding up the onset of complications like neuropathy, nephropathy, and retinopathy [5-6]. HbA1c is a reliable marker of average blood glucose levels over the past 8-12 weeks and a predictor of diabetes-related complications. Magnesium deficiency is common in T2DM because of low dietary intake and increased urinary loss. This deficiency exacerbates insulin resistance, impairs glucose uptake, and is associated with metabolic syndrome [7].

A systematic review highlights a bidirectional relationship between T2DM and hypomagnesemia [8]. Numerous studies show an inverse relationship between serum magnesium levels and blood glucose levels, including fasting blood sugar (FBS) and HbA1c [9-10]. Additionally, a retrospective study linked low serum magnesium levels to other metabolic abnormalities, such as high LDL cholesterol, hypertension, and obesity [11]. A meta-analysis reports a high prevalence of hypomagnesemia among patients with T2DM [12]. There is also evidence that magnesium supplements may help improve overall metabolic control in this group [13].

Although evidence suggests an association between lower serum magnesium levels and poorer glycemic control, most studies are cross-sectional or retrospective, limiting our understanding of this link [9-11]. Specific data from Kerala are also limited, making it hard to apply findings from other populations. This case-control study aimed to provide focused regional evidence to fill this gap in the literature.

The primary objective of this study was to compare serum magnesium levels between adults with uncontrolled and controlled T2DM. The secondary objectives were to examine the correlation of serum magnesium with HbA1c, FBS, lipid profile (total cholesterol, triglycerides, LDL, HDL), BMI, and blood pressure.

## Materials and methods

A case-control study was conducted in the Department of Medicine, Azeezia Institute of Medical Sciences, Kerala, between November 2022 and March 2024, to compare serum magnesium levels between adults with uncontrolled and controlled T2DM and to assess the correlations of serum magnesium levels with HbA1c and other clinical and biochemical variables.

The sample size was calculated using the formula for comparing means between two independent groups, assuming a pooled standard deviation (SD) of serum magnesium of 0.29 mg/dL, at a 5% level of significance (two-sided) and 90% power, using an equal allocation ratio (1:1). This yielded a minimum requirement of 34 participants per group, giving a total sample size of 68.

A total of 68 adult T2DM patients (aged ≥18 years) were enrolled. Consecutive eligible patients attending the outpatient department of medicine during the study period were screened against the inclusion and exclusion criteria and included in the study. Patients were classified as having uncontrolled T2DM (HbA1c >7%, n = 34) or controlled T2DM (HbA1c ≤7%, n = 34) based on the 2021 American Diabetes Association glycemic target guidelines [14]. Cases and controls were frequency-matched by age and sex to ensure comparable distributions between the groups.

Inclusion criteria were adult patients aged ≥18 years with T2DM who had a history of diabetes for more than one year. Exclusion criteria were patients with chronic kidney disease (eGFR <60 mL/min/1.73 m²), thyroid disorders (TSH outside the normal reference range), or alcoholism, as well as those receiving diuretics or magnesium supplementation.

Demographic details and medical history were documented. Physical examination included anthropometry and blood pressure, measured in the seated position on the right upper limb using a mercury sphygmomanometer after five minutes of rest; mean systolic and diastolic blood pressure were recorded. BMI was calculated as weight (kg) divided by height squared (m²) and classified per WHO Asian BMI cutoffs.

After a 12-hour overnight fast, venous blood samples were collected for biochemical analysis. HbA1c was measured using high-performance liquid chromatography on a NEOCARD analyzer. Serum magnesium was estimated using the chlorophosphonazo III colorimetric method, with routine internal quality control. FBS was measured using the glucose oxidase-peroxidase method. Lipid profile parameters were measured using standard enzymatic methods, and LDL cholesterol was directly measured. Lipid values were classified according to National Cholesterol Education Program Adult Treatment Panel III guidelines [15]. Hypomagnesemia was defined as serum magnesium <1.7 mg/dL.

Data were analyzed using SPSS Statistics version 25 (IBM Corp. Released 2017. IBM SPSS Statistics for Windows, Version 25.0. Armonk, NY). Normality of continuous variables was assessed using the Shapiro-Wilk test, and homogeneity of variance using Levene's test. Variables that were normally distributed in both groups were compared using an independent-samples Student's t-test and are presented as mean ± SD. Variables that did not follow a normal distribution were compared using the Mann-Whitney U test and are presented as median (interquartile range (IQR)). Qualitative variables were expressed as frequencies and percentages and compared using the chi-square test. As serum magnesium was not normally distributed, Spearman's rank correlation was used to assess its association with clinical and biochemical variables within the case group (N = 34). A p-value <0.05 was considered statistically significant.

The Institutional Ethics Committee of Azeezia Institute of Medical Sciences and Research approved the study (approval number: AEC/REV/2022/42, dated November 8, 2022). All participants provided written informed consent. Confidentiality was maintained throughout the study.

## Results

Baseline demographic and anthropometric characteristics of study participants

The study included 68 participants (34 cases and 34 controls). The mean age was 60.56 ± 10.66 years among cases and 57.26 ± 12.38 years among controls (t = 1.175, p = 0.244), with no statistically significant difference between the two groups. Most participants in both groups were aged 60 years or older (58.8% cases vs 55.8% controls), with a nearly equal sex distribution (males: 52.9% vs 50%; χ² = 0.058, p = 0.808), indicating comparable distributions between the groups.

Regarding BMI, the largest proportion of participants in both groups had a BMI >25 kg/m² (44.1% of cases vs 38.2% of controls), followed by BMI <22.9 kg/m² (32.3% vs 35.3%) and 23-24.9 kg/m² (23.5% vs 26.5%). The mean BMI was comparable between cases (24.83 ± 3.11 kg/m²) and controls (23.76 ± 3.13 kg/m²), with no statistically significant difference observed (t = 1.414, p = 0.162). Table 1 summarizes the distribution of age, sex, and BMI.

**Table 1:** Baseline demographic and anthropometric characteristics of study participants Normality of continuous variables was assessed using the Shapiro-Wilk test, and homogeneity of variance using Levene's test; the independent t-test (equal variances assumed) was applied where these assumptions were met. Categorical variables are presented as N (%) and compared using Pearson's chi-square test; continuous variables are presented as mean ± SD and compared using the independent-samples t-test. A p-value <0.05 was considered statistically significant. BMI: body mass index, N: number of participants, SD: standard deviation

Characteristics	Cases (n = 34)	Controls (n = 34)	Test statistics	p-value
Age group (years)				
≤50	5 (14.7%)	6(17.6%)	-	-
51-59	9 (26.4%)	9 (26.4%)	-	-
≥60	20 (58.8%)	19 (55.8%)	-	-
Mean age (years) ± SD	60.56 ± 10.66	57.26 ± 12.38	t = 1.175	0.244
Gender				
Male	18 (52.9%)	17 (50%)	χ^2^ = 0.058	0.808
Female	16 (47.0%)	17 (50%)		
BMI				
<22.9	11 (32.3%)	12 (35.3 %)	^-^	-
23-24.9	8 (23.5 %)	9 (26.5%)	-	-
>25	15 (44.1 %)	13 (38.2 %)	-	-
Mean BMI (kg/m²) ± SD	24.83 ± 3.11	23.76 ± 3.13	t = 1.414	0.162

Clinical and biochemical characteristics

Clinical and biochemical parameters differed significantly between cases and controls. FBS was significantly higher in cases (median 187.5, IQR 142-204 mg/dL) than in controls (median 120, IQR 110.25-131.75 mg/dL; U = 941.5, p < 0.001), with 79.4% of cases having FBS ≥126 mg/dL compared to only 26.4% of controls (χ² = 19.12, p < 0.001). Both systolic and diastolic blood pressures were significantly higher in cases than in controls (systolic: median 128, IQR 122.5-135.5 vs median 124, IQR 120-128 mmHg; U = 793, p = 0.008; diastolic: median 83, IQR 80-88 vs median 80, IQR 80-80 mmHg; U = 858.5, p < 0.001). HbA1c was significantly higher in cases than in controls (median 8.3, IQR 7.83-9.0% vs. median 6.7, IQR 6.5-6.9%; U = 1156, p < 0.001), consistent with poorer glycemic control in cases, as expected in this study design. Table 2 summarizes the glycemic profile and blood pressure among study participants.

**Table 2:** Glycemic profile and blood pressure among study participants Normality of continuous variables was assessed using the Shapiro-Wilk test; variables did not follow a normal distribution and were compared using the Mann-Whitney U test, with results presented as median (IQR). Categorical variables are presented as N (%) and were compared using Pearson’s chi-square test. A p-value <0.05 was considered statistically significant. BP: blood pressure, FBS: fasting blood sugar, HbA1c: glycated hemoglobin, IQR: interquartile range

Characteristics	Cases (n = 34)	Controls (n = 34)	Test statistics	p-value
FBS (mg/dL)				
≤125	7 (20.6 %)	25 (73.5%)	χ² = 19.12	<0.001
>125	27 (79.4 %)	9 (26.4%)		
Median (IQR)	187.5 (142-204)	120 (110.25-131.75)	U = 941.5	<0.001
Systolic BP (mmHg)				
Median (IQR)	128 (122.5-135.5)	124 (120-128)	U = 793	0.008
Diastolic BP (mmHg)				
Median (IQR)	83 (80-88)	80 (80-80)	U = 858.5	<0.001
HbA1c (%)				
Median (IQR)	8.3 (7.8-9)	6.7 (6.5-6.9)	U = 1156	<0.001

Laboratory parameters

Lipid Profile and Serum Magnesium Levels

Total cholesterol, triglycerides, LDL, and HDL levels did not differ significantly between cases and controls in either categorical or continuous analyses (all p > 0.05).

However, serum magnesium levels were significantly lower in cases than in controls (p = 0.018). Hypomagnesemia (<1.7 mg/dL) was present in 14.7% (5/34) of cases compared with none of the controls (χ² = 5.396, p = 0.02). Median magnesium was 2.1 (IQR 1.9-2.3) mg/dL in cases versus 2.3 (IQR 2.1-2.3) mg/dL in controls (U = 388, p = 0.018). Table 3 summarizes lipid profile and serum magnesium levels among cases and controls.

**Table 3:** Lipid profile and serum magnesium levels among study participants Normality of continuous variables was assessed using the Shapiro-Wilk test. Variables followed a normal distribution in both groups and were compared using the independent t-test; those that did not follow a normal distribution were compared using the Mann-Whitney U test, with results presented as median (IQR). Categorical variables are presented as N (%) and were compared using the chi-square test. A p-value <0.05 was considered statistically significant. HDL: high-density lipoprotein, IQR: interquartile range, LDL: low-density lipoprotein, N: number of participants, SD: standard deviation

Variable	Cases (n = 34)	Controls (n = 34)	Statistical value	p-value
Total cholesterol (mg/dL)				
Normal (<200)	22 (64.7%)	23 (67.6%)	χ² = 0.065	0.797
Elevated (≥200)	12 (35.3%)	11 (32.3%)		
Mean ± SD	198.06 ± 26.60	195.74 ± 28.39	t = 0.348	0.728
Triglycerides (mg/dL)				
Normal (<150)	25 (73.5%)	27 (79.4%)	χ² = 0.326	0.567
Elevated (≥150)	9 (26.5%)	7 (20.6%)		
Median (IQR)	133 (115-149.25)	135.5 (125.25-145)	U = 511.5	0.418
LDL (mg/dL)				
Optimal (<100)	27 (79.4%)	25 (73.5%)	χ^2 ^= 0.326	0.567
Above optimal (≥100)	7 (20.6%)	9 (26.5%)		
Mean ± SD	94.5 ± 18.5	96.8 ± 20	t = 0.49	0.626
HDL (mg/dL)				
Low	20 (58.82 %)	17 (50 %)	χ^2 ^= 0.533	0.465
Normal	14 (41.18 %)	17 (50 %)		
Median (IQR)	42 (40-45)	44(41-47)	U = 438.5	0.087
Serum magnesium (mg/dL)				
Hypomagnesemia (<1.7)	5 (14.7%)	0	χ^2 ^= 5.396	0.02
Normomagnesemia (1.7-2.4)	29 (85.3%)	34 (100%)		
Median (IQR)	2.1 (1.9-2.3)	2.3 (2.1-2.3)	U = 388	0.018

Relationship Between HbA1c and Serum Magnesium

The scatter plot (Figure 1) demonstrates a clear difference between cases and controls. Cases cluster at higher HbA1c values (7.2-10.0%) than controls (5.9-7.0%), while serum magnesium levels show greater variability in cases, suggesting an inverse relationship between HbA1c and serum magnesium.

**Figure 1:**
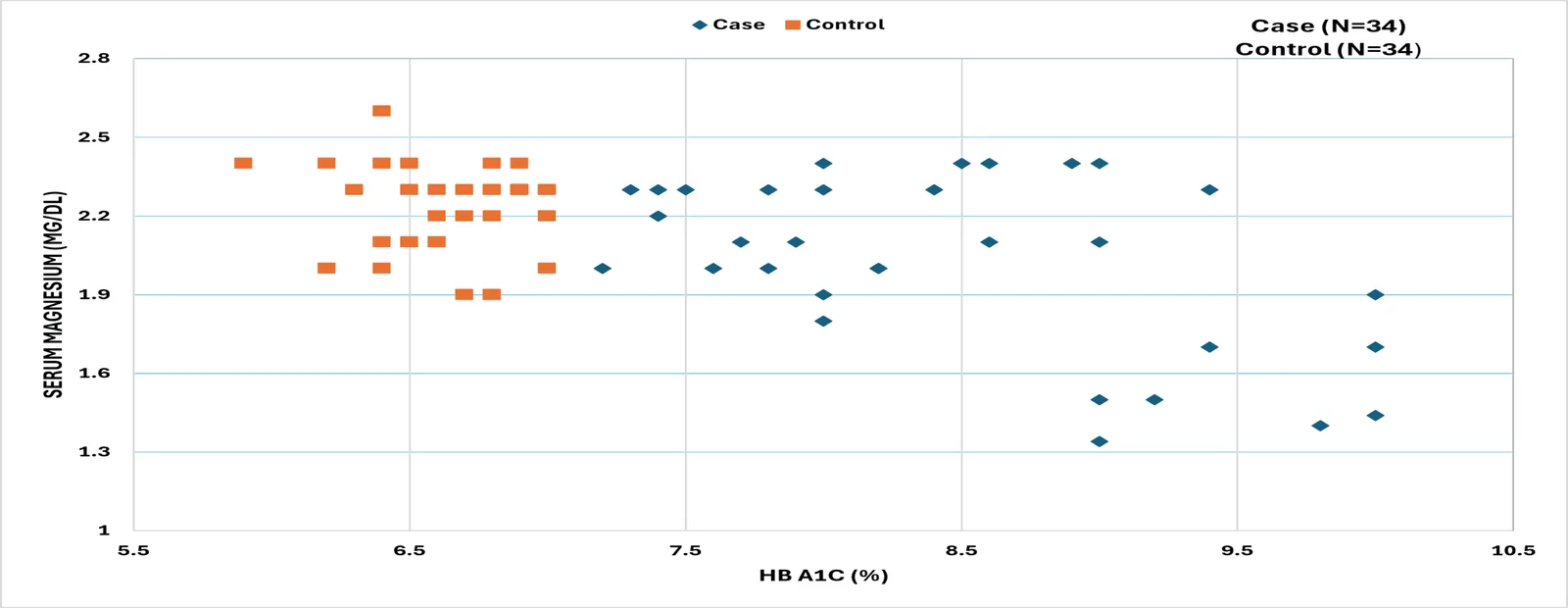
Scatter plot HbA1c vs serum magnesium in cases and controls Each data point represents an individual participant. HbA1c(%): Case >7%, controls ≤7%. Normal serum magnesium range: 1.7-2.4 mg/dL. Cases: diamond, blue. Controls: square, orange. HbA1c: glycated hemoglobin

Correlation of Serum Magnesium With Clinical and Biochemical Variables

Spearman's rank correlation analysis showed that triglycerides had the strongest negative correlation with serum magnesium (ρ = −0.417, p = 0.014), which was statistically significant. Total cholesterol (ρ = −0.335, p = 0.053), LDL (ρ = −0.272, p = 0.120), FBS (ρ = −0.252, p = 0.150), HDL (ρ = −0.237, p = 0.178), HbA1c (ρ = −0.196, p = 0.266), BMI (ρ = −0.125, p = 0.480), diastolic BP (ρ = −0.125, p = 0.482), and systolic BP (ρ = −0.053, p = 0.764) showed progressively weaker negative correlations, but none were statistically significant. Although HbA1c and FBS were inversely correlated with serum magnesium, neither reached statistical significance (Figure 2).

**Figure 2:**
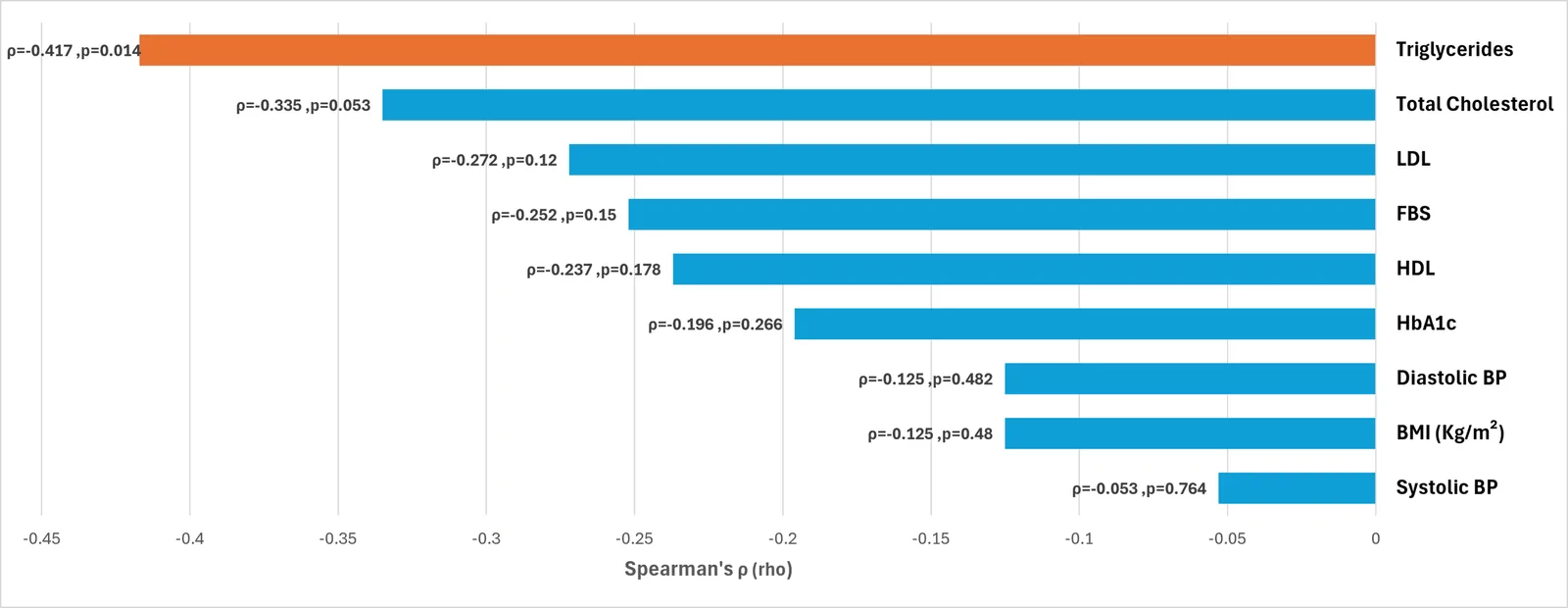
Correlation of serum magnesium with clinical and biochemical variables in cases (N = 34) The bar chart depicts Spearman's rank correlation coefficients (ρ) between serum magnesium and clinical and biochemical variables in the case group. Negative values indicate an inverse relationship with serum magnesium. A p-value of <0.05 was considered statistically significant. ρ: Spearman's rank correlation coefficient, HbA1c: glycated hemoglobin, FBS: fasting blood sugar, LDL: low-density lipoprotein, HDL: high-density lipoprotein, TG: triglycerides, BMI: body mass index, BP: blood pressure

## Discussion

Patients with T2DM frequently have low serum magnesium levels. Low serum magnesium levels in patients with T2DM have been associated with impaired metabolic regulation and increased cardiovascular risk [11]. This case-control study examined the association between serum magnesium levels and HbA1c in patients with T2DM who visited a tertiary care hospital in Kerala.

The socio‑demographic characteristics of cases and controls were comparable, with no significant differences in age, sex, or BMI, suggesting comparable baseline distributions of these characteristics between groups. A substantial proportion of participants were over 60 years old, consistent with the higher prevalence of T2DM in older adults, nearly half of whom are aged ≥65 years globally [15,16]. The mean BMI in both groups was within the overweight range according to the WHO Asian Indian cutoffs (24.83 and 23.76 kg/m², respectively), which is consistent with the characteristic adiposity phenotype observed in Asian Indians, who may exhibit greater central adiposity and increased cardiometabolic risk at comparatively lower BMI levels [17].

FBS and HbA1c were significantly higher in cases, confirming poor glycemic control. Blood pressure was also elevated, consistent with the high prevalence of hypertension among T2DM patients, with reviews from Southeast Asia reporting rates above 70% [18]. Chronic hyperglycemia is thought to drive this association through insulin resistance, oxidative stress, and endothelial dysfunction [19].

In this study, none of the lipid parameters (total cholesterol, triglycerides, LDL, or HDL) differed significantly between cases and controls, in contrast to prior reports that often highlight dyslipidemia in poorly controlled T2DM [11,19]. This may reflect the relatively homogeneous study population.

Hypomagnesemia was significantly more prevalent among cases, supporting the established link between poor glycemic control and hypomagnesemia in T2DM. This bidirectional relationship (hyperglycemia causing urinary magnesium loss and deficiency worsening insulin resistance) is well documented [9-12]. Further studies are needed to clarify the role of magnesium monitoring in T2DM.

In the present study, serum magnesium showed a weak, nonsignificant inverse correlation with HbA1c among patients with uncontrolled T2DM. The direction of this association is consistent with existing literature reporting an inverse relationship between magnesium and glycemic control in diabetic populations [9,20-21], although our data did not reach statistical significance. Magnesium plays a vital role in glucose phosphorylation and insulin receptor signaling, and chronic magnesium deficiency can reduce the activity of enzymes such as tyrosine kinase and promote insulin resistance [7-8]. These mechanisms provide a plausible biological basis for a link between low magnesium and poorer glycemic control.

Similarly, serum magnesium levels showed a nonsignificant inverse correlation with FBS, likely because of the relatively small sample size, whereas larger retrospective studies reported significant correlations [21].

Among the lipid parameters, serum magnesium showed a significant inverse correlation with triglycerides and a borderline inverse correlation with total cholesterol. No significant correlation was found with LDL and HDL. Reports on the magnesium-lipid relationship have been inconsistent, possibly because diabetes itself can alter the relationship between magnesium and lipid metabolism [22].

No significant correlations were observed between serum magnesium and BMI, systolic blood pressure, or diastolic blood pressure. These findings may reflect the similar anthropometric characteristics of the participants and the limited sample size, whereas larger multivariate analyses have shown independent associations [11].

This study has several limitations. The single-center design and small sample size limit generalizability, and multicenter studies with larger cohorts are needed. Several correlations were examined without correction for multiple comparisons, so isolated findings, such as the association with triglycerides, should be interpreted cautiously. Unmeasured variables, including dietary magnesium intake, physical activity, and medication history, may influence the observed associations. The absence of follow-up data also prevents assessment of the long-term impact of hypomagnesemia on glycemic outcomes and diabetic complications.

## Conclusions

This study showed a significant difference in serum magnesium levels between cases and controls. Hypomagnesemia was observed exclusively among uncontrolled T2DM patients, which reinforces the known link between magnesium depletion and poor glycemic control. Among patients with uncontrolled T2DM, serum magnesium levels were significantly inversely correlated with triglyceride levels but showed nonsignificant inverse correlations with HbA1c and FBS. These findings are hypothesis-generating and do not establish causality. Future multicenter studies with larger sample sizes are needed to clarify the relationship between serum magnesium and glycemic control.
